# Sphenopalatine ganglion stimulation with one acupuncture needle for moderate-severe persistent allergic rhinitis: study protocol for a multicenter randomized controlled trial

**DOI:** 10.1186/s13063-015-0707-0

**Published:** 2015-04-23

**Authors:** Lu Zhang, Lei Li, Da-Zhuo Shi, Lu-Quan Chen, Kai-Min Zheng, Kai Cheng, Ye Tao, Hai-Yan Guo, Shu-Liang Li, Jing Liu, Feng Xu, Jian-Wu Shen

**Affiliations:** Xiyuan Hospital, China Academy of Chinese Medical Sciences, No. 1 Xiyuan Caochang Road, Haidian District, Beijing, 100091 China; Beijing Tongren Hospital, Capital Medical University, No. 2, Chongwenmennei Street, Dongcheng District, Beijing, 100730 China; Beijing Baiwan Chinese Medical Clinic, No. 25 Baiwanzhuang Road, Xicheng District, Beijing, 100037 China; Beijing Dacheng Acupuncture Hospital, No. 39 Yuanda Road, Haidian District, Beijing, 100097 China; China Academy of Chinese Medical Sciences, No. 16 Dongzhimenneinanxiaojie, Dongcheng District, Beijing, 100700 China

**Keywords:** Study protocol, Randomized controlled trial, Sphenopalatine ganglion, Persistent allergic rhinitis, Acupuncture, Traditional Chinese medicine

## Abstract

**Background:**

Allergic rhinitis is a symptomatic allergic disease of the nose that affects 10 to 20% of the global population. Chinese otolaryngologists use one acupuncture needle to stimulate the sphenopalatine ganglion because of its potential advantages for treating moderate-severe persistent allergic rhinitis compared with traditional Chinese acupuncture (verum acupuncture); however, little evidence is available to support the wide clinical use thus far. Therefore, we propose a protocol for a parallel, multicenter, assessor-blinded, randomized controlled trial to evaluate sphenopalatine ganglion stimulation with one acupuncture needle compared to verum acupuncture for treatment of moderate-severe persistent allergic rhinitis.

**Methods:**

In the trial, 96 patients previously diagnosed with moderate-severe persistent allergic rhinitis and meeting all inclusion criteria will be allocated to one of two equal therapeutic groups by using a computer-generated randomization list. The interventional group will receive sphenopalatine ganglion stimulation with one acupuncture needle for 4 weeks (once or twice weekly, total four to eight sessions); attending physicians will decide whether the second session is required in a week by examining signs and symptoms. The control group will receive individualized verum acupuncture for 4 weeks (twice weekly, total eight sessions). Follow-up evaluations will be performed 1 month later. The primary outcome measure is the change in the total nasal symptom score from the baseline to week 4. The secondary outcome measures include onset time and duration of effectiveness in every session, change in number of days with moderate-severe persistent allergic rhinitis from the baseline to week 8, change in total immunoglobulin E level and eosinophil count in venous blood from the baseline to week 4, change in Rhinoconjunctivitis Quality of Life Questionnaire score from the baseline to week 4, and clinical waiting time.

**Discussion:**

The trial should provide evidence for the benefits of sphenopalatine ganglion stimulation with one acupuncture needle for treating moderate-severe persistent allergic rhinitis, including better change in total nasal symptom score, faster onset time, longer duration of effectiveness, and shorter treatment time.

**Trial registration:**

Current Controlled Trials: ISRCTN21980724 (registered on 27 March 2014).

**Electronic supplementary material:**

The online version of this article (doi:10.1186/s13063-015-0707-0) contains supplementary material, which is available to authorized users.

## Background

Allergic rhinitis is a symptomatic disorder of the nose induced by immunoglobulin E (IgE)-mediated inflammation of the nasal membranes after allergen exposure [1] and can be classified into intermittent and persistent allergic rhinitis (PER). It has a reported prevalence of approximately 10 to 20% globally and 11.1% in China [2]. Allergic rhinitis causes major illness and disability worldwide and reduces the quality of life and productivity regardless of ethnicity, gender, and age [3]. Some patients develop asthma, further increasing medical and social burdens.

Conventional medical treatments for PER include H1-antihistamines, glucocorticosteroids, leukotriene antagonists, decongestants, anticholinergics, and specific immunotherapy [4]. However, several patients experience side effects, become dissatisfied, and seek complementary and alternative treatments [5,6]. In traditional Chinese medicine, physicians have effectively used verum acupuncture to treat allergic rhinitis for many years [7,8]. Interestingly, a large case study suggested that sphenopalatine ganglion stimulation with one acupuncture needle, a technique developed by a Chinese otolaryngologist and applied in more than 130,000 Chinese patients [9], offers potential advantages with regard to nasal symptoms, onset time, duration of effectiveness, and quality of life.

A recent pilot nonrandomized study (ten patients per group) revealed significantly improved total nasal symptom score (TNSS [10]) (*P* <0.05), total non-nasal symptom score (TNNSS [11]) (*P* <0.05), and Rhinoconjunctivitis Quality of Life Questionnaire (RQLQ [12,13]) score (*P* <0.001) after sphenopalatine ganglion stimulation with one acupuncture needle than after verum acupuncture. After each session, the onset time of effectiveness was shorter (*P* <0.001) and duration of effectiveness was longer (*P* <0.001). Further, patients had fewer symptomatic days one month after sphenopalatine ganglion stimulation (*P* <0.001). On the basis of these results, we propose a protocol for a parallel multicenter assessor-blinded randomized controlled trial to evaluate sphenopalatine ganglion stimulation with one acupuncture needle compared to verum acupuncture for treatment of moderate-severe PER.

## Methods

### Ethics

The study design and methodology adhere to the principles of the Declaration of Helsinki and have been approved by the Xiyuan Hospital Ethics Committee (December 31, 2013; approval number 2013XL062-2). The trial is registered in the ISRCTN register (Current Controlled Trials) as ISRCTN21980724. Each participant will receive an explanation regarding the study protocol and written consent will be obtained. All participants will receive documentation regarding the study protocol and are free to withdraw at any time.

### Study setting

The study will be simultaneously conducted at Xiyuan Hospital, Beijing Tongren Hospital, Beijing Baiwan Chinese Medical Clinic, and Beijing Dacheng Acupuncture Hospital, Beijing, China. It will run from January 2014 to October 2015.

### Sample size

We used the symptomatic outcome of the pilot study (10:10 cases in each group) to calculate the sample size in PASS 2008 software (NCSS, Kaysville, UT). The means for the score of TNSS, serially assessed during the 4-week treatment period, was 4.2071 ± 2.58702 after sphenopalatine ganglion stimulation and 7.4393 ± 2.0368 after verum acupuncture. For a power of 0.9 to detect a significant difference (α = 0.01, two sided), 15 participants per group are required. Forty cases will be enrolled in consideration of four centers’ research capabilities. Allowing for a 20% dropout rate, 48 participants will be enrolled in each group, yielding 96 patients for the study.

### Recruitment

Patients will be recruited from the outpatient clinics of the four participating hospitals. Posters describing the required population, free blood and allergic testing of eligible patients, and contact information of the researcher will also be displayed in the clinics. Moreover, television advertisements will be used to publicize the recruitment.

### Baseline assessment

During the run-in period (baseline), the physical sign score (measured at least twice), TNSS and TNNSS (measured once daily), RQLQ score (measured at least once), and total IgE level and eosinophil count in venous blood will be determined.

### Inclusion criteria

To be eligible, participants should be previously diagnosed with moderate-severe PER, according to the Allergic Rhinitis and Its Impact on Asthma (ARIA) criteria, and meet the following requirements:

1. Have had PER for more than 4 days/week, and more than 4 consecutive weeks, with a disease course of more than 1 year.
2. Age range from 18 to 60 years.
3. Completed AR baseline questionnaire and provided written informed consent.
4. Physical sign score ≥1 and symptom score ≥4.

### Exclusion criteria

Patients will be excluded if they meet the following criteria:

1. Acute sinusitis, active asthma, or diagnosis or suspicion of pneumonia.
2. Nasal abnormalities or rhinopolypus (polypoid lesions can be included).
3. Consumed antihistamines, anticholinergics, corticosteroids, decongestants or antibiotics within 2 weeks before enrollment.
4. Received systemically administered corticosteroids within 6 months or special immune therapy within 1 year before enrollment.
5. Received an alternative and complementary modality, that is, acupuncture or herbal medication, for treatment of AR within 2 months before enrollment.
6. Pregnancy or planning for pregnancy.
7. Serious medical conditions such as uncontrolled hypertension, diabetes mellitus requiring insulin injection, past or current malignant tumor, severe dyslipidemia or liver and kidney dysfunction, anemia, active pulmonary tuberculosis, other infectious diseases, or systemic diseases that cannot be treated by acupuncture.
8. Heavy smoker.

### Randomization and blinding

This is a multicenter prospective, randomized controlled clinical trial. The central randomization will be executed by the Xiyuan Hospital, which will use block randomization to generate the random allocation sequence and prepare predetermined computer-made randomization opaque sealed envelopes. The envelopes will be numbered consecutively and connected into a strain. Each envelope will be separated from the strain and then opened in sequence only after the run-in period when the participants are registered in the trial. Outcome assessors and personnel who deal with the data collection and data analysis will be blinded throughout the entire trial. The acupuncturist and patients cannot be blinded because of the nature of the two different acupuncture techniques in this trial, but the physicians are trained not to communicate with the participants or outcome assessors regarding the treatment procedures and responses. To ensure that all practices at each of the four hospitals are the same, physicians who will enroll participants and assessors who will collect data in the four hospitals will be asked to have a 3-day training seminar concerning treatment modalities and trial documentation prior to the trial. Periodic check-ups on the practices will be performed in each hospital.

### Procedure

**Figure 1:**
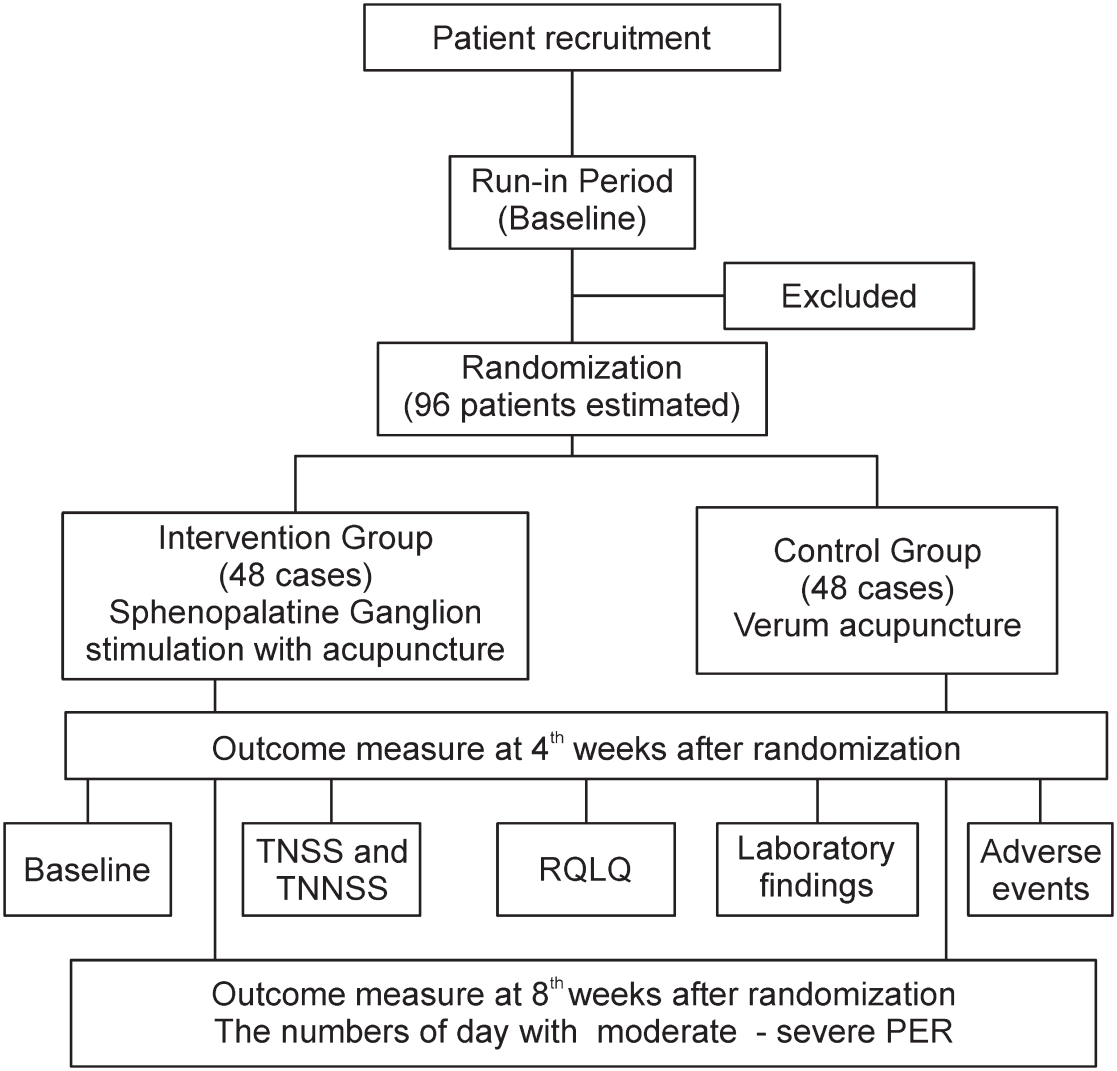
CONSORT flow diagram.

Based on the predetermined randomization envelopes, participants will be randomly allocated to the intervention group or control group (Figure 1). The patients will have an equal probability of being assigned to either of the two groups. Participants will be asked to record symptoms in a rhinitis diary from the run-in period to the 4th week after randomization, and the usage of acute symptomatic relief medication will be recorded during the treatment period. The case report form contains all of the outcome measures and the rhinitis diary will be collected separately from the four hospitals in the 4th week after randomization by blinded interviewers. Blinded telephone interviewers will contact the participants regarding the days of moderate to severe allergic rhinitis during the 4 weeks after the treatment period to evaluate the long-term effect of acupuncture in the 8th week after randomization.

### Interventions

Participants will undergo a standardized interview and receive further information regarding the study. Acupuncturists for the control group must have over 10 years of clinical experience and an acupuncture license issued by the Ministry of Health of the People’s Republic of China. In addition to these criteria, acupuncturists for the interventional group must have been trained by Professor Li Xinwu, the inventor of the sphenopalatine ganglion stimulation technique; have relevant neuroanatomic knowledge; be able to perform the technique clinically; have practiced no fewer than 10 times under Professor Li Xinwu’s supervision; and have practiced independently no fewer than 2,000 times. Before the trial, all acupuncturists will receive special training in the purpose and procedures of the trial, therapeutic strategies, and quality control.

Patients will receive 4 weeks of treatment. In the interventional group, the acupuncturists will use one disposable sterile acupuncture needle (0.35 mm diameter, 60 mm length; Beijing Zhongyan Taihe Medicine Co., Beijing, China) to stimulate the sphenopalatine ganglion. After local disinfection, the needle will be gradually inserted between the zygomatic arch and the coronoid process of the mandible to a depth of approximately 55 mm to enter the pterygopalatine fossa. An additional picture file shows this in more detail [see Additional file 1]. Once the needle touches the sphenopalatine ganglion and the patient experiences a special sensation (radiating toward the nose), the patient will signal with the hand and the needle will be withdrawn immediately. The sphenopalatine ganglion will be stimulated unilaterally in a session. Patients will receive 1 or 2 sessions a week, but most will need only 1 weekly session. The physician will decide whether another session is required on the basis of physical signs and symptoms during the second visit of the week.

The control group will undergo 2 sessions of verum acupuncture per week. The acupoints, including the main and adjunct points (Table 1), have been selected on the basis of Chinese medicine guidelines for allergic rhinitis [14] and are described according to standard nomenclature [15]. Two main and two adjunct points will be applied according to each patient’s symptoms. In particular, 32-gauge (0.25 mm diameter, 25 mm length) sterile needles (Beijing Zhongyan Taihe Medicine Co.) will be used for facial, head, and back points, whereas 30-gauge (0.3 mm diameter, 40 mm length) sterile needles (Beijing Zhongyan Taihe Medicine Co.) will be used for limb and lumbar points. The depth of insertion will vary between 10 and 30 mm (details in Table 1). The acupuncturists will manually manipulate the acupuncture needles until *de-qi* (that is, sensation of tenseness around the needle felt by the practitioner and numbness, distension, soreness, and heaviness around the acupoint felt by the patient) and maintain the needle position for 25 minutes.

**Table 1:** **Verum acupuncture points and techniques for moderate-severe persistent allergic rhinitis**

**Acupoint**	**Direction**	**Depth (mm)**
Main		
Yingxiang (LI20), both	Transversely along the nasolabial sulcus and toward the root of the nose	10 to 15
Yintang (GV29)	Transversely and downward toward the nose	10 to 15
Fengchi (GB20)	Obliquely toward the tip of the nose	13 to 20
Fengfu (GB16)	Perpendicular to the skin	13 to 20
Zusanli (ST36)	Perpendicular to the skin between the tibia and the fibula	20 to 30
Adjunct		
Shangxing (GV23)	Transversely toward the calvarium	8 to 13
Hegu (LI4)	Perpendicular to the skin	13 to 25
Kouheliao (LI19)	Obliquely toward the tip of the nose	5 to 8
Feishu (BL13)	Obliquely toward the spine	13 to 20
Pishu (BL20)	Obliquely toward the spine	13 to 20
Shenshu (BL23)	Perpendicular to the skin	15 to 25
Sanyinjiao (SP36)	Perpendicular to the skin	13 to 25

### Outcome measures

The primary outcome measure is the change in TNSS between the baseline (week 0) and week 4. The secondary outcome measure include the change in TNNSS between the basline (week 0) and week 4, onset time and duration of effectiveness in every session, change in the number of symptomatic days from the baseline to the end of the follow-up (week 8), change in total IgE level and eosinophil count in venous blood from the baseline to week 4, change in RQLQ score from the baseline to week 4, and clinical waiting time (Table 2).

**Table 2:** **Outcome measurements**

**Week**	**0**	**1**	**2**	**3**	**4**	**5**	**6**	**7**	**8**
	**Baseline**	**Treatment period (4 to 8 session)**							**Follow-up**
Sign informed consent	√								
TNSS	√	√	√	√	√				
TNNSS	√	√	√	√	√				
Physical sign	√	√	√	√	√				
RQLQ score	√	√	√	√	√				
IgE level	√				√				
Eosinophil count	√				√				
Number of days with moderate-severe PER in the last month	√								√
Allergen test	√								
Randomization	√								
Onset time		√	√	√	√				
Duration of effectiveness in every session		√	√	√	√				
Waiting time		√	√	√	√				
Adverse event		√	√	√	√				

TNSS, Total nasal symptom score; TNNSS, Total non-nasal symptom score; RQLQ, Rhinoconjunctivitis Quality of Life Questionnaire.

Onset time is the timing of changes in the physical sign score and nasal congestion degree. The physical sign score will be calculated from the degree of inferior nasal concha swelling [16] before and after each session. The nasal congestion degree will be scored using a visual analog scale [17] before and after each session. The duration of effectiveness will be recorded as the duration of change in TNSS after each session (once the patient feels symptom alleviation).

### Data collection

During the study, data will be collected before and after each session and in weeks 0, 4 and 8. The physical sign score will be calculated by outcome assessors approximately 10 to 18 times (week 0, before and after each session), and the RQLQ score will be subjectively determined at least five times (once a week). The total IgE level and eosinophil count in venous blood will be measured in weeks 0 and 4.

Participants will be asked to record symptoms from the baseline to week 4 in a rhinitis diary. They will be required to note the use of relief medication (cetirizine dihydrochloride film-coated tablets) prescribed by the physicians for acute symptoms of allergic rhinitis, including dosage, ingestion time, relief time, and side effects. The case report form containing data for outcome measures and the rhinitis diary will be collected separately from the hospitals by blinded interviewers in week 4. Blinded telephonic interviewers will contact participants regarding the number of moderate-severe symptomatic days with PER up to week 8. Participants will also be asked to report any adverse events including discomfort, bruising, or subcutaneous hematoma around the insertion site; nausea; or feeling faint after each treatment.

Compliance and reasons for discontinuation will be documented in the case report form until week 8. In dropout cases, participants will be contacted by telephone or mail to ascertain the reasons and record the time of the last treatment and last assessment. For withdrawal caused by adverse events or dissatisfaction with the treatment, participants will receive other suitable treatments.

### Statistical analysis

Data will be analyzed by the following principles: (i) intention-to-treat analysis (participants who have at least one measurable outcome after treatment; missing data will be replaced according to the principle of last observation carried forward) and (ii) and per-protocol analysis (participants who have completed the study). A variance analysis will be performed to reject the global hypothesis that “There is no difference in all fields of means between the groups.” The significance level is 5%; therefore *P* <0.05 indicates a significance difference.

The means of TNSS and TNNSS will be compared by analysis of variance (ANOVA) for repeated measures between the groups. Mean onset time and duration of effectiveness will be compared by *t*-test between the groups. Analysis of variance (ANOVA) for repeated measures will be used for between-group comparisons of RQLQ scores. The other continuous variables (total IgE level and eosinophil count in venous blood, number of symptomatic days, and clinical waiting time) will be compared by *t*-test. Comparisons within groups will be performed by multivariate ANOVA followed by Tukey’s post hoc test. Stratified analysis within the four hospitals will be performed to control for confounding factors, if necessary. The incidence of adverse events will be calculated and compared between the groups by using the chi-squared test or Fisher’s exact test. All analyses will be performed in SPSS version 13.0 (SPSS, Chicago, IL).

## Discussion

Sphenopalatine ganglion stimulation with one acupuncture needle requires knowledge of not only traditional Chinese medicine theory but also neuroanatomy and neurophysiology. Furthermore, it is relatively difficult to study and apply clinically. Considering the proven therapeutic efficiency of verum acupuncture for PER, clinical application of a new technique will be unwarranted if evidence for its advantages cannot be obtained.

The proposed randomized controlled trial will investigate the advantages of sphenopalatine ganglion stimulation with one acupuncture needle over verum acupuncture for treating moderate-severe PER. The trial should show benefits of sphenopalatine ganglion stimulation with an acupuncture needle for treatment of moderate-severe persistent allergic rhinitis, including improvement of the total nasal symptom score, faster onset time, longer duration of effectiveness and shorter treatment time.

Many Chinese acupuncturists and otolaryngologists have discussed the endpoints for our pilot study and concluded that inferior nasal concha swelling and nasal congestion are the most serious challenges in traditional Chinese acupuncture. Further, such treatment does not show clinical effectiveness in a short time. In contrast, sphenopalatine ganglion stimulation with one acupuncture needle can quickly alleviate nasal congestion and inferior nasal concha swelling. Therefore, we have decided to observe the onset time and duration of effectiveness in addition to other outcome measures such as TNSS and TNNSS used in other studies.

Our pilot study revealed that sphenopalatine ganglion stimulation with one acupuncture needle yields significant differences in onset time, duration of effectiveness, TNSS, RQLQ score, and number of post-treatment symptomatic days compared with verum acupuncture. Experts in allergic rhinitis were consulted to decide which endpoints to adopt for sample size calculation. They concluded that changes in TNSS are generally applied, and this scoring system has been validated [18]. Therefore, the mean TNSS was used to calculate the sample size for the trial. Furthermore, the research group consensually decided that TNSS should replace onset time and duration of effectiveness, reported in the ISRCTN registry, as the primary outcome measure of the trial. Only 15 cases per group, total 30, are needed based on the sample calculation. As the small sample size may be increase the risk of bias, we decided to increase the patient number to 40 in each group in consideration of each center’s research capabilities. Allowing a drop-out rate of 20%, 96 participants will be enrolled in the study. All other endpoints are considered as secondary outcome measures. The total IgE level and eosinophil count in venous blood will be measured to investigate the mechanism underlying the effect of acupuncture.

We will measure the clinical waiting time to investigate economic benefits. Sphenopalatine ganglion stimulation with an acupuncture needle requires only approximately 1.5 min and no bed. In contrast, patients spend an average of 2 h to receive acupuncture in Chinese hospitals, including 1.5 h or longer waiting for a bed and 20 min or longer per session.

The proposed trial has several methodological limitations. The acupuncturists and participants cannot be blinded because of the difference in acupuncture techniques and control required while needling. However, the acupuncturists will be trained to not communicate the therapeutic procedures and responses to the participants and outcome assessors. Furthermore, this study will not allow us to determine whether the observed efficacy is caused by placebo effects, intensity of provider contact, or physiological effects of needling. The bias resulting from the lack of blinding cannot be totally excluded. Finally, the sample size is small.

## Trial status

The trial is currently in the recruitment phase.

## Additional file

Additional file 1:
**The insertion point and the Pterygopalatine fossa.** The additional file will present two figures giving more details. (a) insertion point for sphenopalatine ganglion stimulation (the figure is cited from the article: Grégoire et al. [19]). (b) The red arrow points out the Pterygopalatine fossa in the skull.

## Abbreviations

ANOVA: analysis of variance
ARIA: Allergic Rhinitis and Its Impact on Asthma
IgE: immunoglobulin E
PER: persistent allergic rhinitis
RQLQ: Rhinoconjunctivitis Quality of Life Questionnaire
TNSS: Total Nasal Symptom Score
TNNSS: Total Nonnasal Symptom Score
