# Supplementary figures and images for: Sphenopalatine ganglion stimulation with one acupuncture needle for moderate-severe persistent allergic rhinitis: study protocol for a multicenter randomized controlled trial
Source: Trials. 2015 Apr 23;16:183. doi: 10.1186/s13063-015-0707-0 (PMC4426638; doi:10.1186/s13063-015-0707-0)

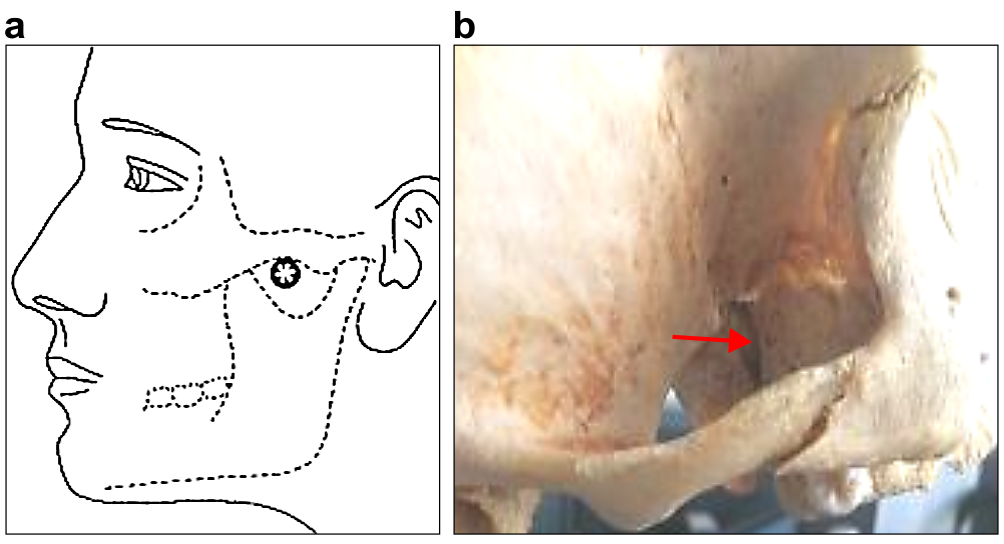

Supplement: Additional file 1: — The insertion point and the Pterygopalatine fossa. The additional file will present two figures giving more details. (a) insertion point for sphenopalatine ganglion stimulation (the figure is cited from the article: Grégoire et al. [19]). (b) The red arrow points out the Pterygopalatine fossa in the skull. [file 13063_2015_707_MOESM1_ESM.tiff]
